# Green Ultrasound-Assisted Extraction of Onion Polyphenols Using a Choline Chloride-Urea Deep Eutectic Solvent: Extraction Efficiency, Solvent Selectivity, and Antioxidant Assay Compatibility

**DOI:** 10.3390/antiox15070826

**Published:** 2026-06-30

**Authors:** Mirjana S. Jankulovska, Raquel Sánchez-Romero, Gabriela Guillena, José Luis Todolí-Torró

**Affiliations:** 1 Faculty of Agricultural Sciences and Food—Skopje, Ss. Cyril and Methodius University in Skopje, P.O. Box 297, 1000 Skopje, North Macedonia; jankulovska@fznh.ukim.edu.mk; 2 Department of Analytical Chemistry, Nutrition and Food Sciences, University of Alicante, P.O. Box 99, 03080 Alicante, Spain; jose.todoli@ua.es; 3 Department of Organic Chemistry, University of Alicante, P.O. Box 99, 03080 Alicante, Spain; gabriela.guillena@ua.es

**Keywords:** polyphenols, deep eutectic solvents (DES), onion, Folin-Ciocâlteu assay, antioxidant activity, ultrasound-assisted extraction, vortex-assisted extraction, UHPLC-MS/MS

## Abstract

Deep eutectic solvents (DES) emerge as sustainable alternatives for antioxidant polyphenol extraction; however, their analytical performance and compatibility with antioxidant assays remain insufficiently characterized. A choline chloride-urea-water DES (1:2:4) was compared with 70% ethanol for extracting polyphenols from onion bulbs and peels from cultivars grown in Spain and North Macedonia. Extraction conditions were selected through a comparative evaluation of vortex- and ultrasound-assisted extraction and benchmarked against conventional ethanolic stirring (2 h). Two-way ANOVA identified solvent composition as the main determinant of total phenolic content (F(1,20) = 1526.28, *p* < 0.001), while extraction method significantly influenced recovery under DES conditions (F(2,30) = 408.52, *p* < 0.001). The selected UAE-DES protocol increased TPC up to 2.2-fold and reduced extraction time to <5 min. TPC ranged from 25 to 44 mg GAE/g dw in bulbs and 42–62 mg GAE/g dw in peels, with red cultivars showing the highest values. UHPLC-MS/MS revealed solvent-dependent selectivity: ethanol favored flavonols (quercetin 5–11 mg/g dw), whereas DES enhanced phenolic acids (gallic acid up to 0.3 mg/g dw; protocatechuic acid up to 7 mg/g dw). FRAP correlated with TPC (r = 0.64–0.92), while ABTS was incompatible with DES extracts. Storage reduced TPC by 45–75% but preserved cultivar ranking. These findings demonstrate that UAE-DES enables rapid and efficient polyphenol recovery while highlighting the need to validate antioxidant assays in non-conventional solvents.

## 1. Introduction

In recent years, increasing attention has been devoted to the functional properties of fruits and vegetables and their contribution to human health, particularly due to their content of bioactive phytochemicals. Among these compounds, polyphenols represent a major class of secondary plant metabolites that significantly influence sensory attributes, nutritional quality, and biological activity of plant-derived foods [1,2]. Polyphenols are of particular interest because of their antioxidant properties and their involvement in mechanisms associated with the prevention or modulation of oxidative stress-related diseases, as extensively documented in the recent literature [3,4,5,6,7]. However, the translation of these biological effects into reproducible food science applications critically depends on reliable extraction, isolation, and characterization strategies.

Among vegetables rich in polyphenols, onion (*Allium cepa* L.) constitutes a relevant and widely consumed matrix for polyphenol research. Onions are particularly rich in flavonoids, mainly quercetin and its glycosylated derivatives, which are among the most abundant and extensively studied polyphenols in this vegetable [8,9,10]. Red onion varieties additionally accumulate anthocyanins responsible for their pigmentation and enhanced antioxidant potential, while phenolic acids such as caffeic and ferulic acid contribute to the overall phenolic profile [11]. Beyond their phytochemical composition, onions are among the most globally cultivated and consumed vegetables, with worldwide production exceeding 100 million tons annually, resulting in sustained population-level dietary exposure to their bioactive constituents [12]. Taxonomically, onions belong to the Amaryllidaceae family and the genus *Allium*, which includes other economically and nutritionally important crops such as garlic, leek, shallot, and chive. In addition to polyphenols, onions contain sulfur-containing compounds and essential mineral nutrients, reinforcing their relevance as a model food matrix for nutritional and phytochemical studies. Consequently, onion polyphenols have attracted increasing interest from both academic research and the food industry due to their potential health-promoting properties [13,14].

Conventional extraction methods based on hydroalcoholic solvents remain widely used due to their simplicity but are increasingly questioned because of environmental concerns, flammability, and limited extraction selectivity [15]. In this context, deep eutectic solvents (DES), composed of biodegradable and tunable components, have emerged as promising green alternatives [16,17]. Their adjustable polarity and extensive hydrogen-bonding networks enable efficient disruption of plant matrices and stabilization of phenolic compounds, frequently resulting in enhanced recovery compared with conventional solvents [17]. Moreover, the strong intermolecular interactions between DES components and phenolic compounds may affect not only extraction efficiency but also the selectivity and composition of the recovered polyphenolic fraction, which is a critical aspect for subsequent characterization [17,18,19,20,21].

Total phenolic content (TPC) is commonly determined using the Folin–Ciocâlteu assay; however, this method estimates overall reducing capacity rather than phenolics exclusively [22,23]. Accurate evaluation of antioxidant activity therefore requires complementary assays, as no single method captures the full spectrum of redox mechanisms. Antioxidant activity is commonly assessed using DPPH (radical scavenging), FRAP (ferric reducing power), and ABTS (electron-transfer capacity) assays, which are widely used to evaluate the bioactivity of food extracts [11,24]. Importantly, solvent composition plays a critical role in both extraction performance and analytical outcomes. While hydroalcoholic solvents generally exhibit limited interference with spectrophotometric assays, DESs may interact with assay reagents, particularly in DPPH and ABTS measurements, thereby affecting result interpretation. In contrast, FRAP has been reported to be less susceptible to solvent-related interference, making it a valuable complementary assay when DES-based extracts are evaluated [24,25,26,27].

The present study was designed as a sequential evaluation of deep eutectic solvent (DES)-based extraction of onion polyphenols. First, the choline chloride:urea:H_2_O DES (1:2:4) was compared with conventional 70% ethanol and extraction conditions were selected using vortex- and ultrasound-assisted extraction. Subsequently, the influence of solvent selection on the qualitative profile of individual phenolic compounds was investigated by UHPLC-MS/MS. The selected extraction procedure was then applied to onion cultivars from different geographical origins to assess its robustness across plant matrices and to evaluate storage stability. Finally, the antioxidant properties of the resulting extracts were examined using complementary assays to assess both extract functionality and the compatibility of antioxidant methodologies with DES-based systems. A schematic overview of the experimental workflow is provided in Figure S1.

## 2. Materials and Methods

### 2.1. Reagents and Samples

The following reagents from Sudelab (Barcelona, Spain) were used: choline chloride, urea, and ethanol. 2,4,6-Triphenyltetrazolium chloride (TPTZ) and iron(III) chloride hexahydrate were obtained from Levantine Laboratories S.L. (Alicante, Spain). Sodium hydroxide (analytical grade; Panreac, Barcelona, Spain) was used to basify the extracts prior to the addition of Folin–Ciocâlteu reagent (Merck, Darmstadt, Germany) for total phenolic content determination.

Gallic acid, Trolox (6-hydroxy-2,5,7,8-tetramethylchroman-2-carboxylic acid), sodium acetate, hydrochloric acid, DPPH (2,2-diphenyl-1-picrylhydrazyl), and ABTS (2,2′-azino-bis(3-ethylbenzothiazoline-6-sulfonic acid)) were purchased from Sigma-Aldrich (St. Louis, MO, USA). Individual polyphenol standards (isorhamnetin, quercetin, kaempferol, ferulic acid, vanillic acid, and protocatechuic acid) were also obtained from Sigma-Aldrich.

### 2.2. Samples and Sample Preparation

Different onion populations (white, yellow, and red) from two geographical origins (North Macedonia and Spain) were used as the study sample. Bulbs were peeled to separate the outer dry skin from the edible tissues. Skins were washed, air-dried, and then milled, whereas the peeled bulbs were oven-dried at 60 °C for 24 h. Dried bulb tissues were subsequently ground using a laboratory blender to obtain a homogeneous powder. All powdered samples were stored in sealed containers protected from moisture and light at room temperature until further analysis.

### 2.3. Deep Eutectic Solvent (DES) Preparation

The deep eutectic solvent (DES) was prepared by combining choline chloride (ChCl) and urea (U) at a 1:2 molar ratio. The required amounts of both components were accurately weighed using an analytical balance, transferred to a reaction flask, and heated under magnetic stirring on an AGIMATIC-N hotplate at 70–80 °C for 60–120 min, until a clear and homogeneous liquid was obtained. Because the resulting ChCl:U DES was highly viscous and could limit mass transfer during extraction, Milli-Q water (Millipore, El Paso, TX, USA) was added at 30% (*w*/*w*). The mixture was stirred until complete homogenization, yielding a hydrated DES with an overall composition of ChCl:U:H_2_O = 1:2:4 (molar ratio). This water content was selected to improve transport properties while preserving the deep eutectic character of the system. In line with previous reports, moderate water addition to choline chloride-based DESs leads to a hydrated DES in which water is incorporated into the hydrogen-bonding network rather than acting solely as a diluent, thereby maintaining dominant ChCl:U supramolecular interactions while substantially reducing viscosity and enhancing extraction efficiency [28,29].

### 2.4. Extraction Procedure Selection

Extraction technique and time were selected using dried yellow onion bulbs from Spain. The solid-to-liquid ratio was fixed at 1:50, following the method of Pal and Jadeja [20]. Briefly, 1.0 g of dried onion powder was mixed with 50 mL of 70% ethanol, whereas 0.2 g was mixed with 10 mL of DES, maintaining the same solid-to-liquid ratio in both cases. Conventional extraction was performed under continuous magnetic stirring for 2 h. In addition, intensified extraction was evaluated using vortex-assisted extraction (VAE) and ultrasound-assisted extraction (UAE) with a probe sonicator operated at 50% and 70% amplitude. Extraction times of 1, 2, 3, 5, and 7 min were tested for both VAE and UAE. All procedures were carried out at room temperature; during UAE, samples were immersed in an ice bath to prevent temperature increases associated with sonication and to minimize potential polyphenol degradation.

To ensure complete phase separation, DES extracts were centrifuged at room temperature (3000 rpm, 15 min). The supernatants were then filtered through 0.45 µm membrane filters prior to analysis. Ethanol extracts were filtered directly without centrifugation. All extracts were stored at 4 °C until analysis. All measurements were performed in triplicate.

### 2.5. Folin-Ciocâlteu Assay

Total phenolic content (TPC) was determined using the Folin-Ciocâlteu colorimetric method [22,23], with slight modifications to accommodate DES-based extracts. An aliquot of 50 μL of appropriately diluted onion extract was mixed with 4750 μL of deionized water and 100 μL of Folin-Ciocâlteu reagent. After 5 min of initial reaction, 100 μL of 30% (*w*/*v*) NaOH was added, and the mixture was vortexed for 1 min. When Folin-Ciocâlteu reagent was added to DES extracts, precipitation was initially observed under conventional alkaline conditions (7.5% Na_2_CO_3_). Therefore, the assay was modified by replacing sodium carbonate with 30% NaOH, resulting in a strongly alkaline medium (pH ≈ 13). This adjustment improved reaction kinetics and minimized precipitate formation, ensuring reliable absorbance measurements for DES-based systems.

For the calibration curve, gallic acid standard solutions were prepared under identical conditions (4800 μL standard solution + 100 μL Folin–Ciocâlteu reagent, followed after 5 min by 100 μL of 30% NaOH). A reagent blank was prepared using 50 μL of extraction solvent instead of sample. All reaction mixtures were incubated in the dark at room temperature for 2 h to allow complete color development. Absorbance was measured at 760 nm using a Biomate-3 UV–VIS spectrophotometer (Thermo Spectronic, Madison, WI, USA). Results were expressed as mg gallic acid equivalents per gram of dry weight (mg GAE/g dw). All analyses were performed in triplicate.

### 2.6. FRAP (Ferric Reducing Antioxidant Power) Assay

The acetate buffer was prepared by mixing 310 mg sodium acetate with 1.6 mL glacial acetic acid and adjusting the final volume to 100 mL with distilled water. The TPTZ solution (2,4,6-tripyridyl-s-triazine) was prepared by dissolving 31.2 mg TPTZ in 10 mL distilled water, followed by the addition of 23.5 μL concentrated hydrochloric acid (HCl, 37% *w*/*w*). The FeCl_3_ solution was prepared by dissolving 54 mg iron(III) chloride in 10 mL distilled water. The FRAP working reagent was freshly prepared by mixing acetate buffer, TPTZ solution, and FeCl_3_ solution in a volume ratio of 10:1:1 (*v*/*v*/*v*). Fresh preparation was required to ensure assay accuracy, sensitivity, and reproducibility.

Aliquots of 200 μL of standard or sample were mixed with 3.0 mL of FRAP reagent. After incubation for 30 min in the dark at room temperature, absorbance was measured at 593 nm using a UV-VIS spectrophotometer. A blank was prepared by mixing 200 μL of the corresponding extraction solvent with 3.0 mL of FRAP reagent. Antioxidant capacity was quantified using Trolox (6-hydroxy-2,5,7,8-tetramethylchroman-2-carboxylic acid) as the reference standard, and results were expressed as μmol Trolox equivalents per gram of dry weight (μmol TE/g dw) [25].

### 2.7. DPPH (2,2-Diphenyl-1-picrylhydrazyl) Assay

A stock solution of DPPH (1 mg/mL) was prepared in absolute ethanol and ultrasonicated for 15 min to ensure complete dissolution. The stock solution was further diluted with ethanol to obtain an initial absorbance of 1.0 ± 0.1 at 515 nm. The assay was performed according to established protocols [30,31]. Aliquots of 200 μL of sample or standard were mixed with 3.0 mL of the diluted DPPH solution. The reaction mixture was incubated in the dark for 30 min at room temperature to allow completion of the radical scavenging reaction. Absorbance was measured at 515 nm using a UV-VIS spectrophotometer. Then, the percentage of DPPH radical scavenging activity was calculated. Antioxidant capacity was quantified using Trolox as the reference standard, and results were expressed as μmol Trolox equivalents per gram of dry weight (μmol TE/g dw) based on calibration curves constructed from the relation between percentage inhibition and Trolox concentration.

### 2.8. ABTS (2,2′-Azino-bis(3-ethylbenzothiazoline-6-sulfonic acid)) Assay

The ABTS assay evaluates antioxidant capacity by monitoring the reduction in the blue-green ABTS radical cation (ABTS^•+^). The ABTS^•+^ working solution was generated by oxidizing ABTS [2,2′-azino-bis(3-ethylbenzothiazoline-6-sulfonic acid)] with potassium persulfate, followed by incubation in the dark for 12–16 h at room temperature to allow complete radical formation. The resulting ABTS^•+^ solution was then diluted with ethanol to an absorbance of 0.70–1.00 at 734 nm. For the assay, 200 μL of sample extract or Trolox standard was mixed with 3.0 mL of the diluted ABTS^•+^ solution. After incubation at room temperature for 5–10 min in the dark, the decrease in absorbance was measured at 734 nm using a UV-VIS spectrophotometer. Then, the percentage of inhibition was calculated. Antioxidant capacity was quantified using Trolox calibration and expressed as μmol Trolox equivalents per gram of dry weight (μmol TE/g dw) [27].

### 2.9. UHPLC-MS/MS Analysis

Individual polyphenols in onion extracts were quantified by UHPLC-MS/MS using an Agilent 1290 Infinity UHPLC System coupled to an Agilent 6490 triple quadrupole mass spectrometer (Santa Clara, CA, USA) equipped with an Agilent Jet Stream ion source in negative ionization (NI) mode. Chromatographic separation of analytes was performed on an Agilent Poroshell 120 EC-C18 column, 3 × 100 mm, 2.7 µm, which was maintained at 25 °C during the analysis [32]. The mobile phase consisted of solvent A (0.1% acetic acid in water) and solvent B (0.1% acetic acid in acetonitrile). The gradient program was as follows: 0 min, 10% B, 6 min, 35% B; 10 min, 45% B; 12.0 min, 10% B for column re-equilibration. The flow rate was set at 0.50 mL/min and the injection volume was 2 µL for all samples [32].

Detection was performed in multiple reaction monitoring (MRM) mode by monitoring specific transitions from precursor ions to their most intense product ions. Instrumental parameters were optimized for maximum sensitivity and signal stability. Source conditions were gas temperature of 275 °C, gas flow 11 L/min, nebulizer pressure 45 psi, capillary voltage 3500 V, fragmentor voltage 380 V, and cell acceleration voltage 4 V. Both quadrupoles were operated at unit resolution (0.7 FWHM), and the dwell time for each transition was 10 ms. For each analyte, the most abundant and selective MRM transition was selected for quantification, while secondary transitions were used for confirmation. Collision energies were individually optimized to achieve maximum product ion intensity. The specific MRM transitions used for quantification of each analyte and the collision energy are summarized in Table S1 [32].

MassHunter Workstation software (Agilent Technologies, Santa Clara, CA, USA; version B.07.01) was used for instrument control and data acquisition. Data processing was performed using MassHunter Qualitative Analysis (version B.07.00) and MassHunter Quantitative Analysis (version B.07.00). For each analyte, the most intense MRM transition was selected as the quantifier ion, while additional transitions were monitored as qualifier ions for confirmation [32].

### 2.10. Statistical Analysis

All measurements were performed in triplicate and results are expressed as mean ± standard deviation (SD). Statistical analyses were conducted using SPSS software (version 28.0, IBM Corp., Chicago, IL, USA).

For extraction evaluation experiments, factorial designs were applied. The effects of solvent type and extraction time (Solvent × Time) under vortex-assisted conditions were evaluated using two-way analysis of variance (ANOVA). Similarly, the effects of extraction method (VAE-DES, UAE-DES 50%, UAE-DES 70%) and extraction time (Method × Time) were assessed by two-way ANOVA when only DES-based systems were considered. When significant effects were detected, pairwise comparisons were performed using Tukey’s honestly significant difference (HSD) post hoc test at a significance level of *p* < 0.05.

For comparisons involving multiple cultivars, tissues (bulb vs. peel), or storage time, one-way ANOVA was applied, followed by Tukey’s HSD test to identify statistically significant differences among groups (*p* < 0.05).

To compare selected extraction conditions with the conventional reference method (70% ethanol, 2 h stirring), multiple comparisons against a single control were conducted using Dunnett-type contrasts with Holm correction to control for family-wise error rate.

Pearson correlation analysis was performed to evaluate the relationships between total phenolic content (TPC) and antioxidant activity (FRAP, DPPH, ABTS). Linear regression equations and correlation coefficients (*r*) were calculated to describe these associations.

## 3. Results

### 3.1. Selection of DES-Based Extraction Conditions

**Figure 1:**
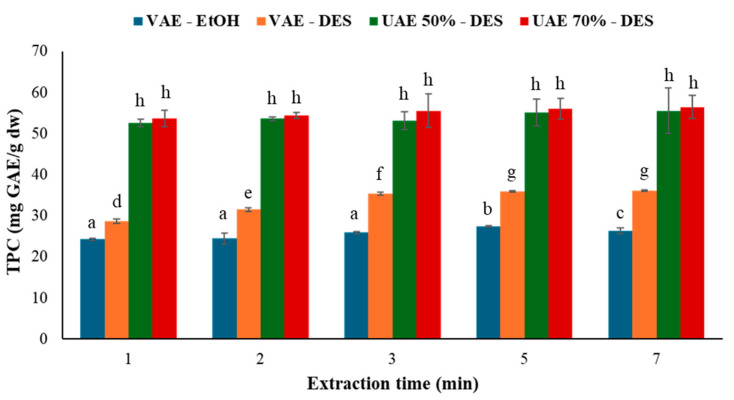
Total phenolic content (TPC) as a function of extraction solvent, extraction technique, and extraction time. Conditions: vortex-assisted extraction with 70% ethanol (VAE-EtOH), vortex-assisted extraction with DES (VAE-DES), and ultrasound-assisted extraction with DES at 50% and 70% amplitude (UAE-DES 50% and UAE-DES 70%). Bars represent mean ± SD (*n* = 3). Different letters indicate significant differences according to Tukey’s HSD test (*p* < 0.05). TPC is expressed as mg GAE/g dw.

As a first step, a comparative screening of solvent type, extraction technique, and extraction time was performed to select suitable working conditions for subsequent analysis of onion samples. Polyphenols from yellow onion bulbs cultivated in Spain were extracted using either 70% (*v*/*v*) ethanol or a deep eutectic solvent (DES) composed of choline chloride:urea:H_2_O (1:2:4). Three extraction approaches were evaluated: conventional magnetic stirring for 2 h (reference method), vortex-assisted extraction (VAE; 1–7 min) using ethanol or DES, and ultrasound-assisted extraction (UAE; 1–7 min) using DES at 50% and 70% amplitude. The conventional extraction yielded a TPC of 24.7 ± 0.7 mg GAE/g dw. The results obtained under the different experimental conditions are summarized in Figure 1. All intensified extraction approaches resulted in higher TPC values than the conventional method, although the magnitude of improvement strongly depended on both solvent and extraction technique.

To distinguish between solvent effects and extraction-technique effects, the experimental evaluation was conducted in two stages. First, ethanol and DES were compared under identical vortex-assisted extraction conditions. Subsequently, the effect of ultrasound-assisted extraction was evaluated within the DES system. In the first stage, VAE using ethanol (VAE-EtOH) was directly compared with VAE using DES (VAE-DES) to isolate the effect of solvent composition under equivalent extraction conditions. Two-way ANOVA (Solvent × Time) revealed a highly significant main effect of solvent (F(1,20) = 1526.28, *p* < 0.001; partial η^2^ = 0.987), indicating that solvent selection accounts for nearly all explainable variance in TPC within vortex-assisted systems. Extraction time also showed a strong effect (F(4,20) = 104.52, *p* < 0.001; partial η^2^ = 0.954), and the interaction term was significant (F(4,20) = 23.59, *p* < 0.001; partial η^2^ = 0.825), demonstrating that the advantage of DES increases with extraction time. Across all evaluated time points, DES yielded systematically higher TPC values than ethanol, with improvements ranging from approximately 20% to 40%.

In the second stage, the analysis was restricted to DES-based systems in order to evaluate the additional contribution of extraction intensification. Two-way ANOVA (Method × Time) showed a highly significant main effect of extraction method (F(2,30) = 408.52, *p* < 0.001; partial η^2^ = 0.964), whereas extraction time contributed to a lesser but still significant extent (F(4,30) = 6.04, *p* = 0.001; partial η^2^ = 0.446). The interaction between both factors was not statistically significant (*p* = 0.358), indicating that the relative performance of the extraction techniques remained stable across time.

Under VAE-DES conditions, TPC increased progressively from 28.6 ± 0.6 mg GAE/g dw at 1 min to 36.04 ± 0.11 mg GAE/g dw at 7 min, reflecting a gradual improvement consistent with time-dependent mass transfer. In contrast, UAE-DES resulted in a markedly different extraction profile, with TPC values exceeding 50 mg GAE/g dw within the first minute. This sharp increase highlights the strong intensification effect associated with ultrasound-assisted extraction. Both ultrasound amplitudes (50% and 70%) significantly outperformed vortex-assisted extraction (Tukey’s HSD, *p* < 0.05). However, no statistically significant differences were observed between both amplitudes, suggesting that increasing acoustic energy beyond 50% did not translate into further gains in phenolic recovery under the conditions tested.

When compared with the conventional reference extraction, all intensified methods yielded significantly higher TPC values. Specifically, VAE-EtOH showed a modest increase (25.65 mg GAE/g dw; *p* = 0.016), whereas VAE-DES resulted in a more substantial improvement (33.48 mg GAE/g dw; *p* = 5.40 × 10^−8^). The highest values were obtained using UAE-DES at both amplitudes (54.23–55.06 mg GAE/g dw; *p* < 10^−15^), corresponding to an increase of up to 2.20-fold relative to the reference method. Importantly, this improvement was achieved while reducing extraction time from 120 min to less than 5 min, demonstrating a clear intensification of the extraction process. Based on these results, UAE at 70% amplitude for 1 min was selected as the working extraction condition for subsequent analyses, as it provided the highest TPC values under the experimental conditions evaluated.

### 3.2. Influence of Solvent Selection on Polyphenol Profiles

To evaluate whether solvent selection affected not only total phenolic recovery but also the qualitative composition of the extracts, individual phenolic compounds were quantified by UHPLC-MS/MS in onion bulbs and peels extracted with 70% ethanol and DES under the selected working conditions. The quantified compounds included gallic acid (GA), protocatechuic acid (PA), vanillic acid (VA), ferulic acid (FA), quercetin (Q), kaempferol (K), and isorhamnetin (I) (Table 1 and Table 2).

Marked solvent-dependent differences were observed. Ethanolic extracts were characterized by higher concentrations of flavonols, particularly quercetin [7]. In peel extracts, quercetin reached 4.7 ± 0.4 mg/g dw in white peel, 10.9 ± 0.5 mg/g dw in yellow peel, and 8.55 ± 0.19 mg/g dw in red peel. Kaempferol and isorhamnetin followed the same trend, showing substantially higher concentrations in ethanol than in DES extracts (Figure S2). These results confirm that hydroalcoholic extraction favors flavonol-rich profiles in onion matrices.

In contrast, DES extraction preferentially enhanced the recovery of phenolic acids. Gallic acid increased in all tissues when DES was used, reaching 0.108 ± 0.007 mg/g dw in white peel, 0.107 ± 0.004 mg/g dw in red peel, and 0.278 ± 0.011 mg/g dw in yellow peel. Protocatechuic acid was also recovered more efficiently with DES in several peel samples, particularly in white peel (1.79 ± 0.08 mg/g dw) and yellow skin (6.9 ± 0.8 mg/g dw), compared with 0.886 ± 0.014 and 5.0 ± 0.4 mg/g dw, respectively, in ethanolic extracts. Vanillic acid showed sample-dependent behavior, with higher values under DES in some samples, such as red skin, but not across all tissues. Ferulic acid did not show a consistent enhancement with DES, indicating compound-specific extraction behavior even within the phenolic acid group.

### 3.3. Application of the Developed Extraction Method to Onion Cultivars and Storage Stability

The selected DES-based extraction procedure was subsequently applied to onion samples from Spain and North Macedonia to evaluate whether the trends observed during the initial extraction assessment were maintained across cultivars of different color and geographical origin (Table 3). This step was not intended to establish cultivar-specific optimal extraction conditions, but to assess the applicability of the selected DES-based procedure to a broader set of onion matrices.

Total phenolic content (TPC) values showed substantial variability depending on both cultivar and geographical origin, ranging from 37.1 ± 0.8 to 61 ± 2 mg GAE/g dw, using the selected extraction method. In all cases, red onion cultivars exhibited systematically higher TPC values than yellow varieties within the same geographical origin. For instance, Spanish red onions reached values up to ~48 mg GAE/g dw under DES conditions, whereas yellow cultivars remained below ~40 mg GAE/g dw. A similar trend was observed for North Macedonian samples, where red cultivars showed the highest TPC values in the dataset (up to 61 mg GAE/g dw).

Geographical origin also contributed to the observed variability. On average, onion samples from North Macedonia showed higher phenolic content than those cultivated in Spain when cultivars of comparable type were considered. This effect was consistent across both extraction methods, suggesting that environmental factors associated with cultivation conditions influence phenolic accumulation. Despite this intrinsic variability, DES extraction consistently yielded higher TPC values than ethanol across all evaluated samples and conditions (Table 3). The magnitude of this increase varied among samples but was systematic, supporting the applicability of the selected UAE-DES procedure across different onion matrices.

The effect of storage was also evaluated by analyzing the same samples after 10 months under controlled conditions (25 ± 2 °C, protected from light). A pronounced decrease in TPC was observed across all samples, with reductions ranging from approximately 45% to 75% depending on cultivar and origin. For example, Spanish yellow onions decreased from initial values around 17–25 mg GAE/g dw to approximately 6–15 mg GAE/g dw after storage, while red cultivars showed similar proportional reductions despite higher initial values. Although absolute TPC values decreased substantially, the relative ranking among cultivars was preserved after storage. Red onions remained the richest source of phenolics, followed by yellow varieties, and the differences between geographical origins were maintained. This indicates that, while storage significantly affects quantitative phenolic content, it does not alter the underlying compositional hierarchy among samples.

### 3.4. Total Phenolic Content and Antioxidant Activity Evaluation for Real Samples

Although total phenolic content is widely used as an indicator of antioxidant potential, the Folin-Ciocâlteu assay does not specifically quantify phenolics but rather estimates total reducing capacity, as the reagent also reacts with non-phenolic reductants such as ascorbic acid, sugars, and aromatic amines. Moreover, antioxidant capacity depends not only on total concentration but also on the molecular structure, substitution pattern, and redox properties of individual phenolics. Consequently, extracts with similar TPC values may exhibit different antioxidant responses. For this reason, complementary assays based on distinct reaction mechanisms, namely FRAP (single-electron transfer reducing power), DPPH and ABTS (radical scavenging assays), were employed to obtain a broader evaluation of antioxidant capacity [33,34].

After applying the selected extraction conditions to real onion samples, total phenolic content and in vitro antioxidant capacity were evaluated in bulb and peel extracts to assess the functional response of the obtained extracts and the compatibility of antioxidant assays with DES-based systems. Extracts from white, yellow, and red onions cultivated in Spain and North Macedonia were analyzed (Table 4 and Table 5). The data confirmed that onion peels generally represented a richer source of antioxidant phenolics than bulbs, irrespective of geographical origin. In ethanolic extracts, bulb TPC ranged from 8.6 ± 0.6 to 16.3 ± 0.4 mg GAE/g dw, whereas peel extracts reached up to 39.7 ± 0.5 mg GAE/g dw. This distribution is consistent with the accumulation of flavonols in the outer dry scales of *Allium cepa* L. [7,35]. DES extraction generally increased TPC values in bulbs and in most peel samples, reaching 23.3 ± 0.5 mg GAE/g dw in bulbs and 53.59 ± 0.16 mg GAE/g dw in peels. An exception was observed for white peel from North Macedonia, where the DES extract showed lower TPC than the corresponding ethanolic extract. The increase in TPC was generally accompanied by higher FRAP values in DES extracts, particularly in red peel samples, where FRAP exceeded 600 μmol TE/g dw. As shown in Figures S3 and S4 and Table S2, strong correlations were observed between TPC and FRAP, especially in ethanolic extracts (r = 0.9077–0.9289), supporting the predominant contribution of phenolic compounds to reducing power, in agreement with previous reports describing close TPC–FRAP relationships in plant matrices [34].

Radical-based assays showed more complex behavior. DPPH values for peels (Table 5) were generally higher in ethanolic extracts despite lower TPC compared with DES extracts. This divergence suggests that DPPH response was influenced not only by phenolic concentration but also by solvent environment and extract composition. To account for solvent effects, assay blanks containing the corresponding extraction solvent were included in all measurements. In the DPPH assay, the absorbance of the DES blank remained stable over time after addition of the radical solution, indicating that interference from the solvent itself was limited and could be largely corrected through blank subtraction. Nevertheless, differences in solvent viscosity, reaction kinetics, and interactions with co-extracted matrix components may still influence the apparent radical-scavenging response. Similar limitations have been reported for colorimetric radical assays, where absorbance at 517 nm can be affected by pigments and matrix co-extractives [36]. In contrast, the ABTS assay could not be reliably applied to DES extracts. The DES blank exhibited a progressive decrease in absorbance following addition of the ABTS radical, indicating ongoing interactions between the solvent system and the chromophore. Although blank correction was applied, the continuous evolution of the signal suggested that matrix-related interference could not be fully compensated. This behavior indicates that DES components themselves contribute to the reduction in the ABTS radical and confirms the unsuitability of this assay for the investigated DES system Radical stability in ABTS systems has been shown to be influenced by solvent polarity and ionic strength, supporting the incompatibility observed here [37].

Correlation analysis (Figure S3) confirmed strong positive relationships between TPC and antioxidant activity, particularly for FRAP and ABTS in ethanolic extracts (r often > 0.90). Correlations with DPPH were moderate but significant (r = 0.72–0.79), consistent with the greater sensitivity of this assay to solvent-matrix interactions [36]. In DES extracts, correlations remained positive but were generally weaker, especially for FRAP in bulbs (r = 0.6431), suggesting that extraction solvent may modulate the apparent relationship between phenolic concentration and measured antioxidant response (Figure S4).

## 4. Discussion

The present study demonstrates that solvent composition is the primary determinant of polyphenol extraction efficiency. DES improved TPC under equivalent VAE conditions, whereas UAE further intensified extraction within the DES system. This finding is consistent with previous studies reporting the superior performance of deep eutectic solvents compared with conventional hydroalcoholic systems for extracting phenolic compounds from plant matrices [17,18,20].

The enhanced extraction efficiency of the choline chloride–urea DES is likely related to its structured hydrogen-bonding network and tunable polarity, which promote interactions with phenolic hydroxyl groups and facilitate matrix swelling. These properties have been widely described for DES systems, particularly those based on choline chloride, where hydrogen bond donor–acceptor interactions govern solvation capacity and extraction performance [19,21,38]. In addition, the presence of water in the DES composition is known to reduce viscosity while preserving the supramolecular structure, further enhancing mass transfer [28,29].

Beyond extraction yield, the present results highlight a key aspect, namely solvent-dependent selectivity. DES preferentially enhanced the recovery of phenolic acids, whereas ethanol favored flavonols such as quercetin, kaempferol, and isorhamnetin. This behavior is consistent with the role of molecular polarity and structural features in governing solute–solvent interactions. Gallic acid and protocatechuic acid contain both hydroxyl and carboxyl groups, enabling multiple hydrogen-bonding interactions with the chloride anion and urea moieties of the DES [19,39]. Their relatively low molecular weight and higher polarity favor solvation in highly structured, hydrogen-bond-rich environments [21]. By contrast, flavonols such as quercetin, kaempferol, and isorhamnetin possess extended aromatic conjugation and lower aqueous solubility due to π–π stacking and intramolecular hydrogen bonding, which reduces their effective polarity [40]. Hydroalcoholic ethanol, with intermediate polarity and amphiphilic character, may more effectively disrupt hydrophobic interactions within plant tissues and facilitate diffusion and solubilization of these moderately lipophilic aglycones [41]. As shown in previous studies [15,16,34], DES extraction selectively enriches highly polar phenolic acids, whereas ethanol favors flavonol-dominated profiles (Figure S2). Importantly, the variability observed among individual compounds, particularly the inconsistent behavior of ferulic acid, indicates that extraction selectivity could not be predicted solely at the level of compound nature. Instead, subtle differences in molecular structure and matrix interactions appear to play a decisive role, as previously reported in studies on complex plant-derived matrices [26]. The present study focused on a single choline chloride–urea–water DES formulation because this system is widely used in polyphenol extraction. However, DES extraction selectivity is highly dependent on the identity of both the hydrogen-bond acceptor and the hydrogen-bond donor. Alternative DES formulations based on glycerol, lactic acid, organic acids, polyols, or other hydrogen-bond donors may modify polarity, viscosity, hydrogen-bonding capacity, and solvation behavior, potentially improving the recovery of flavonols such as quercetin, kaempferol, and isorhamnetin. Therefore, rational optimization of DES composition should be considered an important future direction for tailoring extraction toward specific phenolic compounds.

The analysis of real samples further confirms that both genetic and environmental factors significantly influence phenolic composition. The higher total phenolic content observed in red onion cultivars is consistent with their enrichment in flavonoids, particularly quercetin derivatives, which are known to be abundant in onion outer layers [7,8,10]. Additionally, environmental conditions such as climate and cultivation practices have been shown to modulate phenolic accumulation in onion varieties [9,42].

The substantial reduction in TPC observed after storage is also consistent with the known instability of phenolic compounds, which are susceptible to oxidative degradation and structural transformations over time. Post-harvest handling and storage conditions have been reported to significantly affect flavonoid content in onions [35], supporting the trends observed in the present study. However, the preservation of cultivar-dependent ranking suggests that relative compositional differences remain stable despite quantitative losses.

A particularly relevant contribution of this work is the identification of solvent-dependent limitations in antioxidant assays. While FRAP showed strong correlation with TPC and minimal solvent interference, radical-based assays such as DPPH and ABTS were clearly more strongly affected by solvent–matrix interactions. These findings are in agreement with previous studies highlighting the methodological limitations of antioxidant assays and the influence of reaction mechanism, solvent polarity, and matrix composition on the measured response [25,33,34]. In particular, the incompatibility of ABTS with DES extracts observed in this study can be explained by the sensitivity of the ABTS radical to solvent environment and ionic strength [27,37]. Similarly, the variability observed in DPPH responses may be related to reaction kinetics and interference from co-extracted compounds, as previously discussed in methodological evaluations of this assay [30,31,36]. These results emphasize that antioxidant evaluation should not be considered independently of extraction conditions. Instead, extraction medium and analytical methods must be treated as an integrated system. This perspective is increasingly recognized in literature, particularly in studies addressing the limitations of spectrophotometric assays in complex food matrices [25]. Future studies should investigate whether dilution of DES extracts and optimization of assay conditions, including buffer composition and pH, can reduce this interference and improve the applicability of the ABTS assay to DES-based extracts.

Beyond analytical considerations, the observed differences in the extraction of individual phenolic compounds may have practical relevance. Phenolic acids and flavonoids differ in antioxidant properties, bioavailability, stability, and potential biological activities, suggesting that the choice of extraction system may influence the functional profile of the obtained extracts. From an application perspective, onion peels represent an abundant agro-industrial by-product and a valuable source of bioactive compounds. The efficient recovery of these compounds using environmentally friendly extraction systems may contribute to biomass valorization strategies and support the development of natural antioxidant ingredients for food, nutraceutical, and related industrial applications [43].

## 5. Conclusions

A comprehensive analytical strategy was implemented to evaluate deep eutectic solvent (DES)-based extraction as a sustainable and selective approach for the isolation and characterization of antioxidant polyphenols in complex onion matrices. Rather than providing universal optimization, the comparative evaluation identified selected working conditions under which DES improved TPC relative to ethanol under equivalent VAE conditions, while UAE further intensified extraction within the DES system. The selected UAE-DES procedure increased TPC up to 2.2-fold compared with conventional 2 h ethanolic stirring and reduced extraction time to less than 5 min.

UHPLC-MS/MS analysis confirmed that solvent choice affected not only total recovery but also extract composition. DES preferentially enhanced phenolic acids, particularly gallic and protocatechuic acids, whereas ethanol favored flavonols such as quercetin. These findings show that solvent selection is a critical parameter when extraction is aimed not only at maximizing yield but also at tailoring the phenolic profile of the obtained extracts.

The application of the selected procedure to onion cultivars from Spain and North Macedonia confirmed its usefulness for comparative profiling of real samples. Red cultivars and peel fractions showed the highest phenolic and antioxidant values, supporting onion peels as promising sources of bioactive compounds. Storage for 10 months substantially reduced TPC, but cultivar-dependent ranking was largely preserved.

Finally, the study highlights that extraction medium and antioxidant assay chemistry must be considered together. FRAP was the most robust assay for DES extracts, DPPH required cautious interpretation, and ABTS was unsuitable because of DES-related interference. The DES-UAE workflow supports greener and faster polyphenol extraction while emphasizing the need to validate antioxidant assays when non-conventional solvents are used.

## Figures and Tables

**Table 1:** Concentration of individual polyphenols in onion cultivars extracted with 70% ethanol using UAE at 70% amplitude for 1 min. Values are expressed as mg/g dw and reported as mean ± standard deviation of three independent determinations (*n* = 3).

Compound	White Bulb	White Peel	Yellow Bulb	Yellow Peel	Red Bulb	Red Peel
GA	0.00070 ± 0.00004	0.0158 ± 0.0009	0.0011 ± 0.0002	0.0243 ± 0.0005	0.00060 ± 0.00010	0.0283 ± 0.0005
Q	0.0190 ± 0.0006	4.7 ± 0.4	0.153 ± 0.011	10.9 ± 0.5	0.0734 ± 0.0012	8.55 ± 0.19
VA	0.00106 ± 0.00011	0.043 ± 0.004	0.00177 ± 0.00005	0.0216 ± 0.0010	0.0023 ± 0.0003	0.0032 ± 0.0005
FA	0.0060 ± 0.0006	0.00764 ± 0.00009	0.0105 ± 0.0011	0.0040 ± 0.0005	0.00213 ± 0.00015	0.00435 ± 0.00012
K	0.00097 ± 0.00015	0.764 ± 0.014	0.00289 ± 0.00003	0.116 ± 0.004	0.000189 ± 0.000006	0.0135 ± 0.0005
I	0.0026 ± 0.0002	0.138 ± 0.009	0.0139 ± 0.0004	0.075 ± 0.004	0.0052 ± 0.0002	0.127 ± 0.005
PA	0.000537 ± 0.000005	0.886 ± 0.014	0.0026 ± 0.0003	5.0 ± 0.4	0.00269 ± 0.00012	2.55 ± 0.14

GA: Gallic acid; Q: Quercetin; VA: Vanillic acid; FA: Ferulic acid; K: Kaempferol; I: Isorhamnetin; PA: Protocatechuic acid.

**Table 2:** Concentration of individual polyphenols in onion cultivars extracted with a choline chloride:urea:H_2_O deep eutectic solvent (1:2:4 molar ratio) using UAE at 70% amplitude for 1 min. Values are expressed as mg/g dw and reported as mean ± standard deviation of three independent determinations (*n* = 3).

Compound	White Bulb	White Peel	Yellow Bulb	Yellow Peel	Red Bulb	Red Peel
GA	0.032 ± 0.003	0.108 ± 0.007	0.058 ± 0.010	0.278 ± 0.011	0.0206 ± 0.0006	0.107 ± 0.004
Q	0.00010 ± 0.00002	0.059 ± 0.008	0.000060 ± 0.000004	0.089 ± 0.011	0.0000932 ± 0.0000012	0.130 ± 0.007
VA	0.00165 ± 0.00014	0.0083 ± 0.0002	0.00169 ± 0.00018	0.0067 ± 0.0007	0.0024 ± 0.0006	0.018 ± 0.003
FA	0.0034 ± 0.0005	0.004904 ± 0.000007	0.00181 ± 0.00017	0.00105 ± 0.00010	0.00061 ± 0.00006	0.00163 ± 0.00012
K	0.000151 ± 0.000004	0.010 ± 0.003	0.000145 ± 0.000002	0.0016 ± 0.0002	0.000149 ± 0.000002	0.000464 ± 0.000004
I	0.00024 ± 0.0002	0.0028 ± 0.0003	0.000212 ± 0.000003	0.000207 ± 0.000005	0.00224 ± 0.00018	0.00065 ± 0.00007
PA	0.00165 ± 0.00013	1.79 ± 0.08	0.00204 ± 0.00014	6.9 ± 0.8	0.0081 ± 0.0005	2.39 ± 0.05

GA: Gallic acid; Q: Quercetin; VA: Vanillic acid; FA: Ferulic acid; K: Kaempferol; I: Isorhamnetin; PA: Protocatechuic acid.

**Table 3:** Total phenolic content (TPC) in onion cultivars from Spain (ES) and North Macedonia (MK), determined immediately after sample drying and after 10 months of storage at 25 ± 2 °C in sealed, light-protected containers. TPC values are expressed as mg GAE/g dw and reported as mean ± standard deviation of three independent determinations (*n* = 3).

Samples	After Sample Drying		After 10 Months Storage	
	EtOH (70%)-Stirring 2 h	UAE-DES 70%	EtOH (70%)-Stirring 2 h	UAE-DES 70%
Yellow onion ES (1)	24.7 ± 0.7	42.0 ± 1.7	11.2 ± 1.1	15.0 ± 0.4
Yellow onion ES (2)	17.1 ± 0.5	38.4 ± 0.6	6.7 ± 0.3	14.3 ± 0.5
Yellow onion ES (3)	17.7 ± 0.9	37.1 ± 0.8	6.4 ± 0.7	14.8 ± 1.0
Red onion ES (1)	27.8 ± 0.4	47.6 ± 1.4	10.4 ± 1.0	17.3 ± 0.9
Red onion ES (2)	31.1 ± 0.8	48 ± 3	10.8 ± 0.7	18.8 ± 0.4
Yellow onion MK (1)	39.1± 1.1	48.7 ± 1.5	14.7 ± 1.2	15.8 ± 1.6
Yellow onion MK (2)	35.3 ± 1.6	49.9 ± 1.3	12.0 ± 0.9	16.0 ± 1.0
Red onion MK (1)	43.8 ± 1.3	56 ± 2	19 ± 2	17 ± 2
Red onion MK (2)	43 ± 2	61 ± 2	13.2 ± 0.7	18.8 ± 1.4

**Table 4:** Total phenolic content (TPC) and in vitro antioxidant capacity of onion bulb extracts from Spain (ES) and North Macedonia (MK). TPC values are expressed as mg GAE/g dw, and antioxidant capacity values are expressed as µmol TE/g dw. Values represent mean ± standard deviation of three independent determinations (*n* = 3). For DES extracts, ABTS values are not reported because of solvent-related interference.

Samples	TPC (mg GAE/g dw)		FRAP (µmol TE/g dw)		DPPH (µmol TE/g dw)		ABTS (µmol TE/g dw)
	EtOH	DES	EtOH	DES	EtOH	DES	EtOH
White bulb MK	8.6 ± 0.6	15.2 ± 0.5	24.1 ± 0.4	60.2 ± 0.6	3.63 ± 0.05	7.7 ± 0.3	80.8 ± 0.5
Yellow bulb MK	11.8 ± 0.3	17.8 ± 0.2	48.7 ± 1.2	65.5 ± 0.6	7.29 ± 0.10	6.82 ± 0.18	138 ± 2
Red bulb MK	16.3 ± 0.4	22.0 ± 0.4	98 ± 3	146.7 ± 1.9	10.41 ± 0.08	12.41 ± 0.19	183 ± 2
White bulb ES	8.8 ± 0.3	16.1 ± 0.3	27.1 ± 1.0	100 ± 7	2.96 ± 0.11	7.50 ± 0.06	83.3 ± 0.9
Yellow bulb ES (1)	12.8 ± 0.3	18.2 ± 0.6	45.3 ± 1.8	136 ± 3	5.94 ± 0.07	8.3 ± 0.2	122.8 ± 0.3
Yellow bulb ES (2)	13.30 ± 0.10	20.3 ± 0.2	51.5 ± 0.3	129 ± 4	4.19 ± 0.11	8.54 ± 0.17	130.0 ± 1.5
Red bulb ES (1)	14.71 ± 0.18	23.3 ± 0.5	76 ± 3	218 ± 6	7.38 ± 0.11	10.0 ± 0.3	167 ± 2
Red bulb ES (2)	15.4 ± 0.2	22.0 ± 0.3	73.2 ± 0.4	184 ± 4	11.09 ± 0.12	12.2 ± 0.3	176 ± 2

**Table 5:** Total phenolic content (TPC) and in vitro antioxidant capacity of onion peel extracts from Spain (ES) and North Macedonia (MK). TPC values are expressed as mg GAE/g dw, and antioxidant capacity values are expressed as µmol TE/g dw. Values represent mean ± standard deviation of three independent determinations (*n* = 3). For DES extracts, ABTS values are not reported because of solvent-related interference.

Samples	TPC (mg GAE/g dw)		FRAP (µmol TE/g dw)		DPPH (µmol TE/g dw)		ABTS (µmol TE/g dw)
	EtOH	DES	EtOH	DES	EtOH	DES	EtOH
White peel MK	8.01 ± 0.19	2.3 ± 0.4	7.8 ± 0.2	14.8 ± 0.3	12.2 ± 0.9	4.4 ± 0.2	8.52 ± 0.14
Yellow peel MK	36.9 ± 0.2	47.2 ± 0.9	468 ± 14	545 ± 11	649 ± 9	176 ± 6	420 ± 9
Red peel MK	38.0 ± 0.4	53.59 ± 0.16	631 ± 7	668 ± 5	737 ± 4	222 ± 3	512 ± 4
White peel ES	17.7 ± 0.5	34.4 ± 0.4	246 ± 15	282 ± 6	288 ± 3	66.2 ± 1.1	230 ± 3
Yellow peel ES (1)	23.6 ± 1.1	47.44 ± 0.19	325 ± 2	427 ± 3	334 ± 2	134 ± 3	294 ± 3
Yellow peel ES (2)	17.7 ± 0.5	43.3 ± 0.7	324 ± 14	412 ± 6	429.5 ± 1.5	118 ± 3	342 ± 3
Red peel ES (1)	39.7 ± 0.5	51.5 ± 1.1	650 ± 3	683 ± 10	469.8 ± 1.0	217 ± 3	532 ± 2
Red peel ES (2)	38.4 ± 0.8	52.0 ± 0.6	597 ± 11	613 ± 9	719 ± 3	168 ± 3	660 ± 3

## Data Availability

Data are contained within the article and Supplementary Materials.

## Supplementary Materials

The following supporting information can be downloaded at: https://www.mdpi.com/article/10.3390/antiox15070826/s1, Figure S1. Experimental workflow of the study. Onion bulbs and peels from different cultivars and geographical origins were subjected to extraction using either 70% ethanol or a choline chloride–urea–water deep eutectic solvent (DES, 1:2:4 molar ratio). Extraction conditions were evaluated through vortex-assisted extraction (VAE) and ultrasound-assisted extraction (UAE), and the selected UAE-DES conditions were subsequently applied to real onion samples. Extracts were characterized by determination of total phenolic content (TPC), UHPLC-MS/MS analysis of individual polyphenols, antioxidant activity assays (FRAP, DPPH, and ABTS), and storage stability evaluation; Figure S2. Ratio of individual phenolic compound concentrations obtained with 70% ethanol relative to those obtained with the choline chloride-urea-water DES (1:2:4). Values represent the mean ± SD of the six onion samples (white, yellow, and red bulbs and peels; n = 6). Ratios >1 indicate higher recovery with ethanol, whereas ratios <1 indicate higher recovery with DES; Figure S3. Linear correlations between total phenolic content (TPC, mg GAE/g dw) and in vitro antioxidant capacity measured by FRAP, DPPH, and ABTS assays (µmol TE/g dw) in onion bulb and peel extracts obtained with 70% ethanol. Each point represents an individual onion sample, including white, yellow, and red cultivars from Spain and North Macedonia. Dotted lines indicate least-squares linear regression fits. Pearson correlation coefficients (r) and regression equations are reported in Table S2; Figure S4. Linear correlations between total phenolic content (TPC, mg GAE/g dw) and in vitro antioxidant capacity measured by FRAP and DPPH assays (µmol TE/g dw) in onion bulb and peel extracts obtained with the choline chloride–urea–water DES (1:2:4). Each point represents an individual onion sample, including white, yellow, and red cultivars from Spain and North Macedonia. Dotted lines indicate least-squares linear regression fits. Pearson correlation coefficients (r) and regression equations are reported in Table S2. ABTS data are not included because of solvent-related interference observed in DES extracts; Table S1. UHPLC-MS/MS transitions and operating parameters used for the quantification of individual phenolic compounds, including compound name, precursor ion (m/z), product ion (m/z), and collision energy (V); Table S2. Linear correlations between total phenolic content (TPC) and in vitro antioxidant capacity in onion bulb and peel extracts. Regression equations (y = ax + b) and Pearson correlation coefficients (r) are reported for FRAP, DPPH, and ABTS assays, stratified by tissue type (bulb/peel) and extraction solvent (70% ethanol or DES). TPC is expressed as mg GAE/g dw, and antioxidant capacity is expressed as µmol TE/g dw. ABTS data are reported only for ethanolic extracts because of solvent-related interference in DES extracts.
