# Supplementary material for: Green Ultrasound-Assisted Extraction of Onion Polyphenols Using a Choline Chloride-Urea Deep Eutectic Solvent: Extraction Efficiency, Solvent Selectivity, and Antioxidant Assay Compatibility
Source: Antioxidants (Basel). 2026 Jun 30;15(7):826. doi: 10.3390/antiox15070826 (PMC13405354; doi:10.3390/antiox15070826)
Supplement: Supplementary file 1 [file antioxidants-15-00826-s001.zip › antioxidants-4291434-supplementary.pdf]

Green Ultrasound-Assisted Extraction of Onion Polyphenols Using a Choline Chloride-Urea Deep Eutectic Solvent: Extraction Efficiency, Solvent Selectivity, and Antioxidant Assay Compatibility

Mirjana S. Jankulovska,[1] Raquel Sánchez-Romero,[2,*] Gabriela Guillena[3], José Luis Todolí-Torró[2]

[1] Ss. Cyril and Methodius University in Skopje, Faculty of Agricultural Sciences and Food - Skopje, P.O. Box 297, 1000 Skopje, North Macedonia
[2] University of Alicante, Department of Analytical Chemistry, Nutrition and Food Sciences, P.O. Box 99, 03080 Alicante, Spain
[3] University of Alicante, Department of Organic Chemistry, P.O. Box 99, 03080 Alicante, Spain
* Correspondence: r.sanchez@ua.es

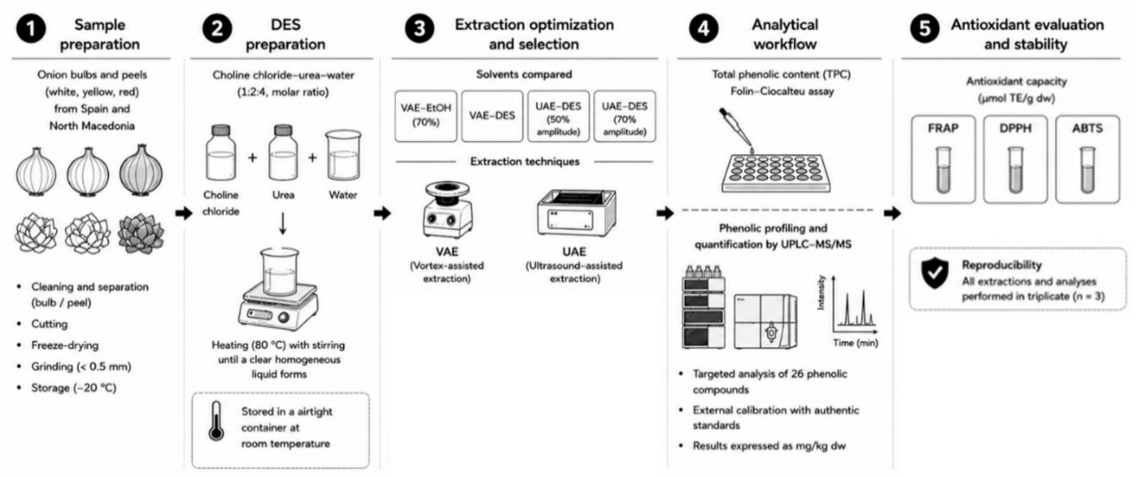

Figure S1. Experimental workflow of the study. Onion bulbs and peels from different cultivars and geographical origins were subjected to extraction using either 70% ethanol or a choline chloride–urea–water deep eutectic solvent (DES, 1:2:4 molar ratio). Extraction conditions were evaluated through vortex-assisted extraction (VAE) and ultrasound-assisted extraction (UAE), and the selected UAE-DES conditions were subsequently applied to real onion samples. Extracts were characterized by determination of total phenolic content (TPC), UHPLC-MS/MS analysis of individual polyphenols, antioxidant activity assays (FRAP, DPPH, and ABTS), and storage stability evaluation.

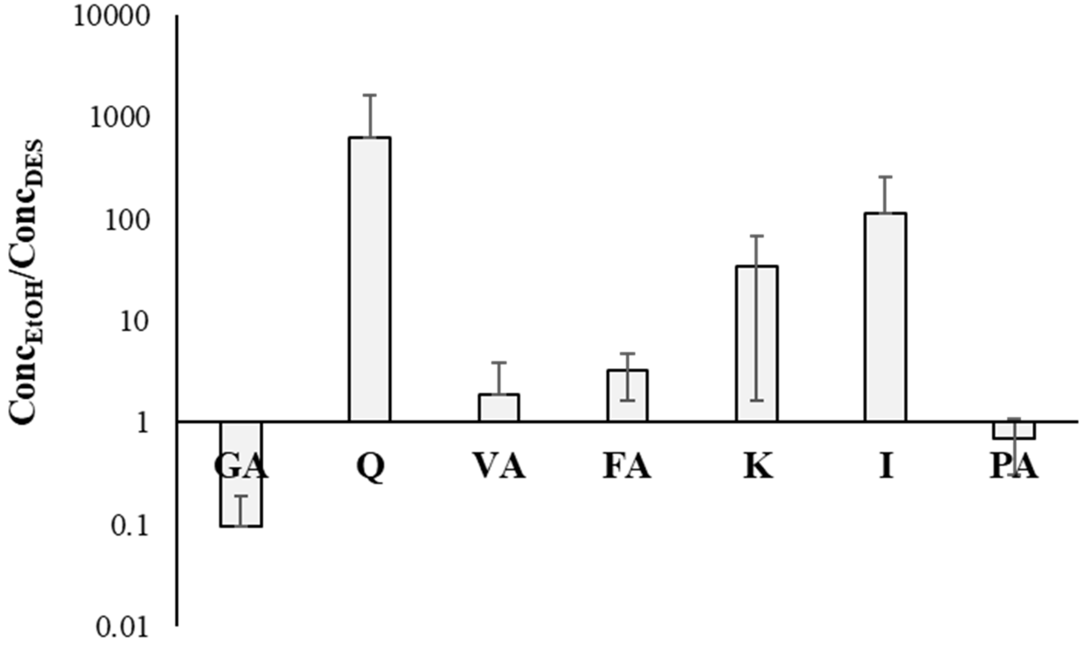

Figure S2. Ratio of individual phenolic compound concentrations obtained with 70% ethanol relative to those obtained with the choline chloride-urea-water DES (1:2:4). Values represent the mean ± SD of the six onion samples (white, yellow, and red bulbs and peels; n = 6). Ratios >1 indicate higher recovery with ethanol, whereas ratios <1 indicate higher recovery with DES.

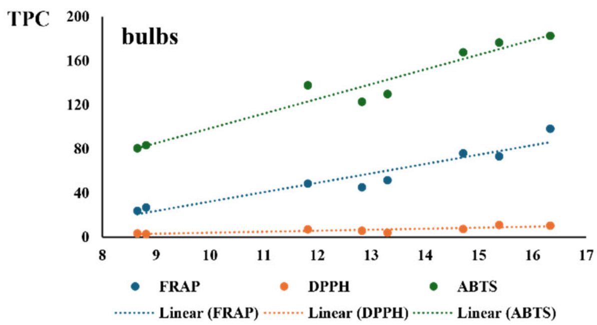

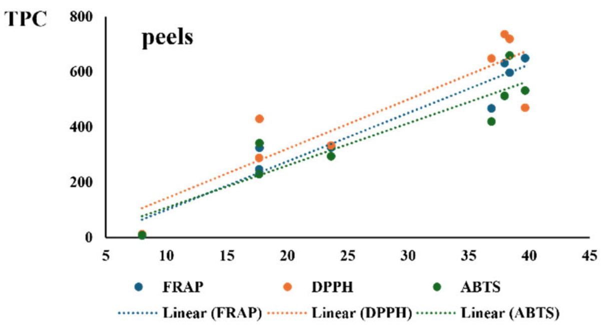

Figure S3. Linear correlations between total phenolic content (TPC, mg GAE/g dw) and in vitro antioxidant capacity measured by FRAP, DPPH, and ABTS assays (μmol TE/g dw) in onion bulb and peel extracts obtained with 70% ethanol. Each point represents an individual onion sample, including white, yellow, and red cultivars from Spain and North Macedonia. Dotted lines indicate least-squares linear regression fits. Pearson correlation coefficients (r) and regression equations are reported in Table S2.

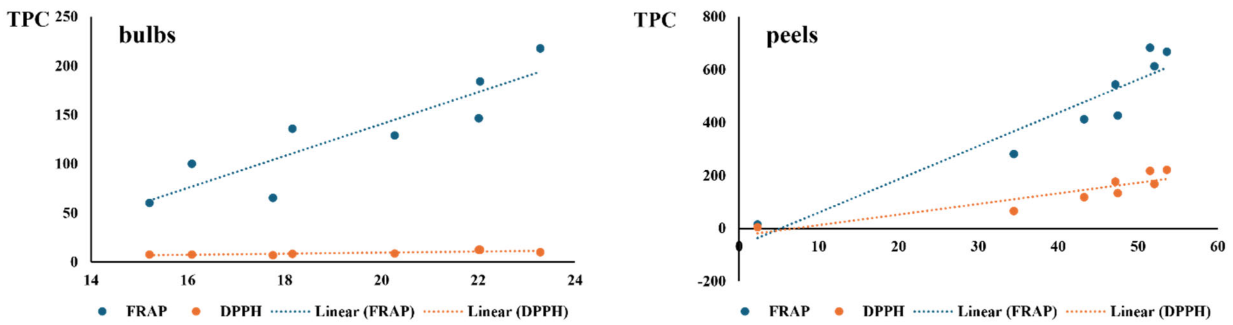

Figure S4. Linear correlations between total phenolic content (TPC, mg GAE/g dw) and in vitro antioxidant capacity measured by FRAP and DPPH assays (μmol TE/g dw) in onion bulb and peel extracts obtained with the choline chloride–urea–water DES (1:2:4). Each point represents an individual onion sample, including white, yellow, and red cultivars from Spain and North Macedonia. Dotted lines indicate least-squares linear regression fits. Pearson correlation coefficients (r) and regression equations are reported in Table S2. ABTS data are not included because of solvent-related interference observed in DES extracts.

Table S1. UHPLC-MS/MS transitions and operating parameters used for the quantification of individual phenolic compounds, including compound name, precursor ion (m/z), product ion (m/z), and collision energy (V).

Compound Name	Precursor Ion (m/z)	Product Ion (m/z)	Collision Energy (V)
Isorhamnetin	317	302	25
Isorhamnetin	317	229	30
Isorhamnetin	317	153	40
Quercetin	303.054	228.8	30
Quercetin	303.054	153.2	30
Kaempferol	287.2	153.3	30
Kaempferol	287.2	121.2	30
Ferulic acid	193.05	178	12
Ferulic acid	193.05	133.9	16
Gallic acid	169.01	125	14
Gallic acid	169.01	51	40
Vanillic acid	167.03	152	12
Vanillic acid	167.03	108.1	20
Protocatechuic acid	153	109.1	10
Protocatechuic acid	153	91.1	30

Table S2. Linear correlations between total phenolic content (TPC) and in vitro antioxidant capacity in onion bulb and peel extracts. Regression equations (y = ax + b) and Pearson correlation coefficients (r) are reported for FRAP, DPPH, and ABTS assays, stratified by tissue type (bulb/peel) and extraction solvent (70% ethanol or DES). TPC is expressed as mg GAE/g dw, and antioxidant capacity is expressed as µmol TE/g dw. ABTS data are reported only for ethanolic extracts because of solvent-related interference in DES extracts.

Sample	Solvent	Correlation TPC – antioxidant activity	Regression equation	*r*
Onion bulbs	EtOH	FRAP	y = 8.4826x – 52.454	0.9077
	EtOH	DPPH	y = 0.9019x – 4.8685	0.7246
	EtOH	ABTS	y = 13.362x – 3.951	0.9412
Onion peels	EtOH	FRAP	y = 17.53x – 75.747	0.9289
	EtOH	DPPH	y = 17.898x – 37.217	0.7896
	EtOH	ABTS	y = 15.295x – 45.499	0.8487
Onion bulbs	DES	FRAP	y = 0.5765x – 1.9643	0.6431
	DES	DPPH	y = 16.291x – 185.33	0.7931
Onion peels	DES	FRAP	y = 3.9843x – 27.13	0.8170
	DES	DPPH	y = 12.551x – 64.967	0.8883
